# Silymarin seed extract supplementation enhances the growth performance, meat quality, and nutrients digestibility, and reduces gas emission in broilers

**DOI:** 10.5713/ab.21.0539

**Published:** 2022-03-02

**Authors:** Sureshkumar Shanmugam, Jae Hong Park, Sungbo Cho, In Ho Kim

**Affiliations:** 1 Department of Animal Resource and Science, Dankook University, Cheonan 31116, Korea

**Keywords:** Blood Profile, Broiler, Meat Quality, Growth Performance, Silymarin

## Abstract

**Objective:**

A feeding trial was carried out to determine the effect of dietary inclusion of silymarin seed extract on growth performance, nutrient digestibility, excreta microbiota, excreta gas emission, blood profiles, and meat quality in broilers.

**Methods:**

A total of 1,088 one-day-old Ross 308 broiler chicks (mixed-sex) with an initial body weight of 42.34±0.82 g, were randomly allocated into 1 of 4 dietary treatments with 17 replicates of 16 chicks per cage and fed a basal diet supplemented with 0%, 0.02%, 0.04%, and 0.06% of silymarin.

**Results:**

The inclusion of silymarin supplementation linearly increased the body weight of broilers during days 7 to 21 and 1 to 35 days. On day 35, broilers fed a diet containing graded levels of silymarin supplementation linearly increased the nutrient digestibility of dry matter, gross energy, and nitrogen and cecal *Lactobacillus* counts (p = 0.038). While silymarin supplement linearly reduced the methyl mercaptans (p = 0.039) and acetic acid (p = 0.007) emission in broilers. No significant effects were observed on the blood profile. Relative weights of organs such as breast muscle, bursa of fabricius were increased (linear effect, p<0.05), water holding capacity was enhanced by increasing the silymarin level from 0% to 0.06%. A linear reduction (p>0.05) in drip loss from meat samples during days 1, 3, 5, and 7 by the addition of graded level of silymarin to the diet.

**Conclusion:**

An increasing level of silymarin supplementation to the diet of broiler would be beneficial to enhance growth performance, nutrient digestibility, excreta microflora, blood profile, and meat quality traits.

## INTRODUCTION

Plants and their extracts have been used for various purposes since ancient period [1]. Besides, they are rich in phytogenics and widely used in livestock diets. For several decades, a number of feed additives including antibiotics as growth promoters (AGP) have been extensively used in livestock feedstuffs. However, the anxieties about antibiotic residues and disease resistance have aroused great caution and lead to the prohibition of the use of antibiotics in animal feed [2]. Consequently, the ban on the use of AGP as feed additives has provoked researchers to find alternative feed additives that could promote the growth performance of animals. Several researches have reported that plant extracts such as quercetin, kalongi (*Nigella sativa*), chicory (*Cichoriumintybus*), neem (*Azadirachtaindica*) yacon (*Smallanthussonchifolius*), and ginger (*Zingiberofficinale*) could be used as an excellent AGP [3,4].

Over the past 2000 years, *Silybum marianum* seeds have been used as a natural drug to cure the liver and biliary duct. *Silybum marianum* (Milk thistle) is a native herb that is a member of the *Carduus marianum* family [4]. It grows in Northern hemisphere countries, Canada, China, Mexico, West Pakistan, and Kashmir, it has reddish-purple flowers with big leaves that are typically thorny. Dried extracts from the milk thistle seeds have approximately 60% silymarin. Silybin (flavonolignan) is the primary bioactive constituent of silymarin. Besides, it has potential antioxidant that helps to prevent lipid oxidation [5]. Furthermore, Chand et al [6] reported that Silymarin exhibits anti-inflammatory, hepatoprotective, cytoprotective, and anticarcinogenic effects. The alternation in the internal homeostasis and oxidant or antioxidant balance of broiler chickens occurs when they are exposed to some sort of stress (e.g.: immune challenges, heat stress, transport, and hatching) that leads to oxidative stress, which can have damaging effects on meat shelf life [7]. However, Tedesco et al [8] had reported the positive effects on feed utilization and consumption in broilers by the addition of silymarin. Even though many studies have reported the positive effects of silymarin in animal and human models, according to our knowledge there were few studies presented on broiler performance. Thus, we attempt this study to use silymarin as a natural growth promoter in broiler diets and evaluate how it enhances the growth performance, meat quality, excreta microbiota, nutrient retention, blood profiles and reduced gas emission.

## MATERIALS AND METHODS

Prior to the trail, experimental procedures were reviewed and approved (DK-1-2032) by the Animal Care and Ethics committee of Dankook University, Cheonan, Republic of Korea.

### Preparation of silymarin extract

Dried *Silybum marianum* seeds were pulverized and sieved through 60 mm-mesh size screen to produce a fine powder from which an ethyl alcohol extract (silymarin) was made. In brief, twenty grams of powder was defatted by soxhlation in three hundred milliliters of petroleum ether for sixteen hours. The defatted powder was then soaked in ethanol (300 mL) for ten hours before being evaporated in a vacuum drying oven (Binder, Tuttlingen, Germany) at 39°C.

### Birds’ husbandry, experimental design and dietary regimen

A total of 1,088 1-day-old (mixed-sex) Ross 308 broiler chicks with an initial average body weight (BW) of 42.34±0.82 g were randomly assigned to one of four experimental diets. The experimental diets were basal diet supplemented with 0%, 0.02%, 0.04%, and 0.06% of silymarin. Each treatment had 17 replications with 16 birds/cage. The starter (d 1 to 7), grower (d 8 to 21), and finisher (d 22 to 35) diets were formulated according to NRC standards (Table 1) [9]. Chicks were reared in three-layer battery cages (1.75 m×1.55 m). The room temperature was kept at 33°C for the first 5 days, then dropped to 24°C 60 percent humidity) until the experiment ended. Throughout the trial, the chicks had unrestricted access to water and mash feed.

### Sampling and clinical analysis

Broilers were fed a nutritious diet for 35 days. At the beginning, days 7, 21, and 35 broilers were weighed on a pen basis. The amount of diet ingested, and residual (pen basis) were recorded to measure feed intake (FI). Body weight gain (BWG), FI, and feed conversion ratio (FCR) were calculated at the end of the trial, and daily mortalities (%) were recorded, and FI data was adjusted to account for the BW of dead birds. From days 28 to 35, the feed was mixed with 0.20% chromium oxide as an indigestible marker to determine nutrient digestibility of dry matter (DM), nitrogen (N), and gross energy (GE). On day 35, fresh excreta samples were grabbed (approximately 50 g) from 2 cages/treatment using the stainless-steel collection tray and stored at −20°C. Then the samples were dried using forced convection oven for72 h, pulverized, and sieved using 1 mm sieve. The DM analysis was done following the published method of Sampath et al [10]. The crude protein (N×6.25) absorption in the samples was determined using a Tecator Kjeltec8400 analyzer (Hoeganaes, Sweden). The GE was determined by measuring heat of combustion in the samples, using a bomb calorimeter (Parr 6100; Parr Instrument Co., Moline, IL, USA). The chromium absorption in the samples was determined using the UV-1201 spectrophotometry (Shimadzu, Kyoto, Japan) and the results were recorded. Nutrient digestibility was calculated using [1− ([Nf×Cd]/[Nd×Cf])]×100, where Nf, nutrient concentration in excreta (% DM); Cd, chromium concentration in diet (% DM); Nd, nutrient concentration in diet (% DM); and Cf, chromium concentration in excreta (% DM).

On day 35, fresh excreta samples which were strewn on the stainless-steel collection tray (2 cages/treatment) were collected using micro-tubes, placed in sterile plastic bags, and taken to the research laboratory. To determine the presence of microorganisms, 1 gm of fresh excreta sample was diluted in 9 mL of 1% of peptone broth, mixed well with a vortex mixer and then 0.1% diluted samples were poured into Salmonella-Shigella, MacConkey, and Lactobacilli medium III agar plates, respectively. The Lactobacilli medium III agar plates were incubated for two days at 39°C whereas, Salmonella-Shigella and MacConkey agar plates were incubated for 1 day at 37°C. Later the colonies (*Lactobacillus*, *Escherichia coli* [*E. coli*], *Salmonella*) were enumerated and log transferred for statistical analysis. On the same day, approximately 300 g of fresh excreta samples were collected from each treatment, pooled well, and placed in an airtight plastic box of 2,600 L with a small aperture on one side. Then the box was secured firmly with adhesive tape and fermented for 7 days at 25°C. Following the fermentation process, the concentration of noxious gas (NH_3_, H_2_S, Methyl mercaptans, CO_2_, and acetic acid) in the excreta sample were measured using the procedures of Nguyen and Kim [11].

At the end of the trial (d 35) blood samples were random ly drawn from the brachial veins of 34 birds/treatment using a sterile syringe and kept in (K3EDTA) (Becton, Dickinson, and Co., Franklin Lakes, NJ, USA) heparinized and non-heparinized tubes. Enzymatic kits (Roche Diagnostics GmbH, Mannheim, Germany) were used to determine low density lipoprotein (LDL)- and high-density lipoprotein (HDL)-cholesterol in an automatic analyzer (Roche Cobas 6000 c501, Tokyo, Japan). An automated biochemical analyzer RA-1000 was used to assess the total cholesterol and triglyceride concentrations in blood samples (Bayer Corp., Tarrytown, NY, USA).

Following blood collection, the broilers were individually weighed, taken to the abattoir, and killed by cervical dislocation. The relative organ weight was determined by weighing each organ separately and calculating the mass BW and belly fat, liver, gizzard, spleen, breast muscle, and bursa of Fabricius were carefully removed. With a portable Konica Minolta CR-400 chroma meter (Konica Minolta, Osaka, Japan), meat color characteristics such as yellowness, redness, and brightness standards of each sample (surface) were evaluated at three places. Broiler meat pH was calculate using the portable pH suspension with two buffers (pH 4.0 and 7.0) and the measurement process was repeated for 2 times. For water holding capacity (WHC) 0.2 gm sample was placed in 125 mm (diameter) filter paper and pressed for 3 min. The moisture-exposure of the compressed areas were determined using a digitalized area-line sensor (MT-10S; M.T. Precision Co. Ltd., Tokyo, Japan). The ratio of water: meat area was then calculated (a smaller ratio indicates increased WHC) and recorded. To estimate cooking loss, 4 gm of breast meat samples was taken, packed into zipper bags, cooked in a water bath for 20 min at 80°C. Later the samples were led to cool down for 1 h. Boiled samples were re-weighed. Finally, the cooking loss was determined by calculating the difference between the raw and boiled sample and noted. Meat samples weighing 4 gm were sliced and weighed. Then sliced samples were placed in a Ziplock bag, stored at 4°C, and weighed on days 1, 3, 5, and 7 respectively. The initial and final weight of each sample were used to determine the drip loss level.

### Statistical analysis

SAS (general linear model procedure) was used to analyze all the experimental data as a complete randomized block design (SAS Inst. Inc., Cary, NC, USA; 2000). The linear, quadratic, and cubic effects were used to investigate the polynomial contrast of increasing dietary silymarin supplementation. For growth performance, nutrient digestibility, and excreta gas emission the cage was used as an experimental unit. The individual chicken was used as the experimental unit for measuring excreta microbiota, and meat quality. Significant results were defined as less than 0.05, while trends were defined as less than 0.10.

## RESULTS

A linear increase in BWG during days 7 to 21 (p = 0.013) and overall period (p = 0.043) was observed as the dietary silymarin supplementation increased from 0% to 0.06%. However, there was no significant effect was observed on the FI, FCR, and mortality rate (%) during the entire experimental period (Table 2). However, the addition of silymarin supplementation had linearly improved the nutrient digestibility of DM, N, and energy (p<0.05) in broilers (Table 3). Also, the graded level of silymarin supplementation in broiler diet had linearly increased the *Lactobacillus* (p = 0.038) population and tendency to reduce *E. coli* counts (p = 0.075) (Table 4). There was a linear reduction in excreta methyl mercaptans and acetic acid emission as the dietary silymarin supplementation increased from 0% to 0.06%. The addition of silymarin in the diet of broilers slightly reduced H_2_S emission while no differences were observed in excreta CO_2_ and NH_3_ emissions (Table 5). No treatment effects were observed on the blood profile (Table 6) of broilers fed diet containing silymarin supplementation. Though there were no statistical differences observed on meat color (lightness [L], redness [a], yellowness [b]), the relative weights of organs such as breast muscle, bursa of fabricius were linearly increased (p<0.05), and the spleen weight was linearly decreased with an increasing level of silymarin supplement. The inclusion of silymarin supplementation from 0% to 0.06% in the diet of broilers linearly increased (p<0.05) the pH level, WHC, and reduced the drip loss of meat from days 1, 3, 5, and 7 (Table 7).

## DISCUSSION

The current findings showed that the dietary inclusion of silymarin supplementation improved the FI and BWG of broilers was correlated with Tedesco et al [8] who observed an increased BWG in broilers fed *Silybum marianum* seed supplementation. Whereas Khaleghipour et al [12] study described that increasing level silymarin supplementation reduced the FI and daily weight gain of Japanese quail fed. Previously, Blevins et al [13] reported that broilers fed a diet containing *Silybum marianum* showed no improvements in their feed efficacy. Likewise, Schiavone et al [14] reported that the addition of silymarin supplementation in broilers diet had no positive effects on their growth performance. Jang et al [15] demonstrated that broilers fed diets containing a high dose of plant extract mixtures (carvacrol, cinnamaldehyde, and capsaicin) showed significantly increased activities of pancreatic trypsin and α-amylase, as well as intestinal maltase, compared with the birds fed control and antibiotic diets. We assume that the probable reason for increased weight gain of the broilers might be due to the presence of growth enhancing properties in silymarin extract. In addition, it is possible to alter the digestibility by changing intestinal microflora. The plant extract gives beneficial microorganisms an advantage in nutrient competition with harmful microorganisms by limiting the growth of harmful microorganisms in the intestines, which can increase the nutrient digestibility of broiler chickens [16]. The number of species, herbs, and plant extracts has been reported to enhance digestibility function by stimulating the secretion of digestive enzymes in the gastric mucosa and increasing nutrient intake in broiler [17]. For instance: Jang et al [15] found that broilers fed a diet rich in plant combinations such as cinnamaldehyde, capsaicin, and carvacrol had considerably higher pancreatic trypsin and -amylase activity, as well as intestinal maltase activity, than control and antibiotic-fed chickens. In this study, the nutrient digestibility of DM, nitrogen, and GE had linearly increased in broilers fed silymarin supplemented diets.

The species and populations of microorganisms in the digestive system have a great impact on gut health [18]. Zhu et al [19] demonstrated that chicken’s digestive tract is not only complicated, but it also contains live microbial communities that could play an important role in promoting their intestinal health. *Lactobacillus* are beneficial intestinal bacteria, whereas, *E. coli* and *Salmonella* bacteria frequently cause gut health issues in young animals. The dietary silymarin supplementation 500 ppm and 1,000 ppm resulted in reduced bacterial counts at both days 28 and 42 in aflatoxin-challenged broiler chicks [20]. As we all know, an increased in *Lactobacillus* and decreased *E. coli* count in broilers results in better intestinal health [21]. In this study, lactic acid bacteria (*Lactobacillus*) were linearly increased, and coliform bacteria (*E. coli*) showed a tendency in reduction by increasing the level of silymarin concentration in the diet. The decreased coliform bacteria count and increased lactic acid bacteria counts may also serve as further evidence to explain the improved broiler weight gain and nutrient digestibility. The most hazardous gases in poultry are ammonia, methane, hydrogen sulfide, and carbon dioxide [22]. The environment, animals’ health, and production are affected by odor emissions from livestock industries and lead to civil complaints [16]. The fecal noxious gas content in pigs was decreased when herbal extract mixture supplementation was included [23]. In our study, methyl mercaptans and acetic acid were significantly reduced. Sharma et al [24] found that supplementing the diet with plant extracts or phytogenic feed additives including saponin and polyphenol reduced volatile chemicals in broiler excreta ammonia gas emission. However, there were no significant differences found in CO_2_ and NH_3_ emission. The inconsistent results regarding excreta noxious gas emission may be due to the difference in dosage levels or animals. The findings on silymarin extract reducing the excreta gas emissions in broiler chickens are limited thus sufficient comparison could not be made.

Metwally et al [25] demonstrated that addition of a silymarin concentrate produced significant decrease the triglyceride, HDL, LDL cholesterol concentrations in rats. However, in the present study, triglyceride, HDL, and LDL cholesterol concentration showed no treatment effects by the inclusion of graded level of Silymarin in the diet. In addition, Banaee et al [26] stated that the activity of silymarin in fish decreases plasma glucose and total cholesterol levels almost completely. Tumova et al [27] noted that the inclusion of silymarin decreases serum cholesterol levels and HDL levels which were slightly higher. In the *in vitro* culture technology using silybin it was found that the intake of 3 hydroxy 3-methylglutaryl co enzyme A reductase, which is an important enzyme in cholesterol synthesis [28] has a possible direct influence on liver cholesterol metabolism. Moreover, Silymarin is related to the decrease in liver cholesterol [29] and this function is mainly due to fat-mediated improved bioavailability and/or by inhibition of resorption of dietary cholesterol. And the birds which were treated with silymarin had reduced concentrations of triglycerides which is in line with Khazaei et al [30] who observed a similar result in Japanese quail. Recently, consumers are very much concerned about the safety and quality of meat. Therefore, it is important to assess meat quality [31]. Hossain et al [32] stated that the pH of meat is directly related to the quality of muscle acid and has an impact on the sheer intensity, drip loss, and color of meat. The present results showed that a significant effect on the pH value, drip loss, and WHC. There is lack of information on the effect silymarin on meat quality, including WHC and natural drip loss in broiler chicken. An important effect that takes place in meat quality is drip loss. Reis et al [33] stated that a higher level of WHC of meat broiler chickens is mainly due to the feed additives which depend on essential oils. Nutrient elevation takes place by water loss which mainly leads to loss of juiciness, tenderness of meat [34]. Balamuralikrishnan et al [35] stated that drip loss was an ordinary indicator and lesser drip loss indicates better meat quality. Previously, Duclos et al [36] reported that the carcass with a higher level of meat yield and with lesser fat indicated a better meat quality. Likewise, Fani Makki et al [37] reported that *Silybum marianum* seed had a significant effect on bursa, spleen, abdominal fat, liver, and gizzard and these results agreed with our study, in which broilers fed 0.06% silymarin supplementation had significantly increased breast meat yields, bursa of fabricius and reduced abdominal fats, and spleen. Due to the lack of literature on the effect of silymarin on the meat quality of broilers further comparisons cannot be made.

## CONCLUSION

Based on the current findings, it can be concluded that increasing levels of silymarin supplementation up to 0.06% to the diet of broilers would be beneficial in improving the growth performance, nutrient digestibility, excreta microflora, blood profile, and meat quality traits.

## Figures and Tables

**Table 1:** Ingredient composition of experimental diets as-fed basis

Items	Starter	Grower	Finisher
Ingredients (%)			
Corn	54.19	55.38	56.77
Soybean meal, 45% CP	33.80	26.1	18.23
Canola meal	5.00	10.0	15.0
Soybean oil	2.10	3.62	5.07
MDCP	-	1.28	1.12
DCP	1.70	-	-
Limestone	1.15	1.34	1.22
L-lysine, 78.4%	0.50	0.65	0.81
DL-Methionine, 99%	0.46	0.47	0.52
L-Threonine, 98.5%	0.20	0.25	0.32
L-Tryptophan, 90%	-	0.01	0.04
NaHCO_3_	0.10	0.10	0.10
Salt	0.30	0.30	0.30
Vitamin premix^1)^	0.20	0.20	0.20
Mineral premix^2)^	0.20	0.20	0.20
Choline	0.10	0.10	0.10
Analyed values (%)			
Metabolizable energy (kcal/kg)	3,000	3,100	3,200
Crude protein	23.0	21.5	20.0
Lysine	1.50	1.40	1.30
Methionine+Cystine	1.08	0.99	0.94
Total phosphate	0.48	0.44	0.41
Calcium	0.96	0.87	0.81

MDCP, monocalcium phosphate; DCP, dicalcium phosphate.

1) Provided per kg of complete diet: 11,025 IU vitamin A; 1,103 IU vitamin D_3_; 44 IU vitamin E; 4.4 mg vitamin K; 8.3 mg riboflavin; 50 mg niacin; 4 mg thiamine; 29 mg d-pantothenic; 166 mg choline; 33 μg vitamin B_12_.

2) Provided per kg of complete diet: 12 mg Cu (as CuSO_4_·5H_2_O); 85 mg Zn (asZnSO_4_); 8 mg Mn (as MnO_2_); 0.28 mg I (as KI); 0.15 mg Se (as Na_2_SeO_3_·5H_2_O).

**Table 2:** The effect of Silymarin supplementation on growth performance of broiler

Items	Silymarin				SEM	p-value	
	
	0%	0.02%	0.04%	0.06%		Linear	Quadratic
d 1–7							
BWG (g)	123	131	126	134	3.24	0.381	0.692
FI (g)	148	156	149	156	3.38	0.339	0.983
FCR	1.184	1.203	1.190	1.168	0.02	0.747	0.467
d 7–21							
BWG (g)	662^b^	663^b^	666^ab^	680^a^	9.18	0.014	0.902
FI (g)	1,006	1,012	1,019	1,038	13.41	0.124	0.712
FCR	1.521	1.529	1.532	1.527	0.02	0.651	0.840
d 21–35							
BWG (g)	945	966	970	991	24.13	0.272	0.932
FI (g)	1,716	1,748	1,752	1,771	27.74	0.240	0.787
FCR	1.818	1.812	1.807	1.795	0.03	0.544	0.979
d 1–35							
BWG (g)	1,729^b^	1,759^a^	1,761^a^	1,805^a^	23.11	0.044	0.995
FI (g)	2,867	2,916	2,920	2,965	30.26	0.076	0.906
FCR	1.658	1.658	1.658	1.645	0.01	0.331	0.964
Mortality rate (%)	0.926	2.778	3.704	3.704	-	0.635	1.000

SEM, standard error of means; BWG, body weight gain; FI, feed intake; FCR, feed conversion ratio.

a,b Means in the same row with different superscripts differ (p<0.05).

**Table 3:** The effect of silymarin supplementation on the nutrient digestibility in broilers

Items	Silymarin				SEM	p-value	
	
	0%	0.02%	0.04%	0.06%		Linear	Quadratic
d 35 (%)							
Dry matter	73.07^b^	72.94^b^	73.81^a^	74.12^a^	1.30	0.036	0.334
Nitrogen	67.24^ab^	65.75^b^	68.39^a^	68.73^a^	1.51	<0.001	0.414
Energy	73.26^ab^	72.60^b^	74.40^a^	74.48^a^	1.09	0.004	0.387

SEM, standard error of means.

a,b Means in the same row with different superscripts differ (p<0.05).

**Table 4:** The effect of Silymarin supplementation on microbes in broilers

Items	Silymarin				SEM	p-value	
	
	0%	0.02%	0.04%	0.06%		Linear	Quadratic
d 35 (log_10_cfu/g)							
*Lactobacillus*	7.60^b^	7.69^ab^	7.87^a^	7.88^a^	0.19	0.038	0.361
*Escherichia coli*	6.16	5.80	5.60	5.62	0.22	0.075	0.327
*Salmonella*	5.23	4.64	4.55	4.60	0.29	0.113	0.232

SEM, standard error of means.

a,b Means in the same row with different superscripts differ (p<0.05).

**Table 5:** The effect of Silymarin supplementation on the gas emission of broilers

Items	Silymarin				SEM	p-value	
	
	0%	0.02%	0.04%	0.06%		Linear	Quadratic
d 35 (ppm)							
NH_3_	13.2	13.0	12.3	11.8	1.8	0.316	0.756
H_2_S	1.1	1.3	1.0	1.1	0.2	0.068	0.512
Methyl mercaptans	7.4^a^	7.4^a^	3.3^b^	4.3^b^	1.6	0.039	0.485
CO_2_	1,225	1,275	1,150	1,000	110	0.104	0.634
Acetic acid	1.4^a^	1.5^a^	1.2^b^	1.2^b^	0.2	0.007	0.903

SEM, standard error of means.

a,b Means in the same row with different superscripts differ (p<0.05).

**Table 6:** The effect of Silymarin supplementation on the blood profile of broilers

Items	Silymarin				SEM	p-value	
	
	0%	0.02%	0.04%	0.06%		Linear	Quadratic
d 35 (mg/dL)							
Cholesterol	116	116	114	114	4	0.229	0.593
Triglyceride	26	32	25	25	3	0.086	0.854
HDL cholesterol	83	83	86	86	4	0.232	0.651
LDL cholesterol	28	27	24	23	4	0.054	0.358

SEM, standard error of means; HDL, high density lipoprotein; LDL, low density lipoprotein.

**Table 7:** The effect of Silymarin supplementation on organ weight and meat quality in broilers

Items	Silymarin				SEM	p-value	
	
	0%	0.02%	0.04%	0.06%		Linear	Quadratic
d 35							
Relative organ weight (%)							
Breast muscle	18.86^b^	19.39^b^	19.41^a^	19.63^a^	0.52	0.023	0.191
Liver	2.55	2.47	2.39	2.40	0.10	0.132	0.473
Spleen	0.14^a^	0.14^a^	0.13^b^	0.13^b^	0.01	0.001	0.051
Abdominal fat	1.12	1.10	0.82	0.81	0.14	0.084	0.854
Bursa of Fabricius	0.17	0.16	0.16	0.18	0.10	0.047	0.315
Gizzard	1.32	1.35	1.23	1.28	0.02	0.122	0.325
Breast muscle color							
Lightness (L*)	52.00	54.85	52.01	53.14	1.16	0.473	0.226
Redness (a*)	12.89	14.43	14.26	14.58	0.69	0.334	0.715
Yellowness (b*)	12.36	15.60	14.45	14.01	1.01	0.592	0.072
pH value	5.51^b^	5.50^ab^	5.62^ab^	5.85^a^	0.10	0.012	0.439
Cooking loss (%)	28.09	28.73	30.98	28.97	1.52	0.505	0.759
WHC (%)	53.62	49.45	54.30	55.20	2.43	0.018	0.788
Drip loss (%)							
Day 1	1.72^a^	2.24^a^	1.68^b^	1.64^b^	0.22	0.001	0.863
Day 3	3.71^ab^	4.01^a^	3.60^b^	3.65^b^	0.29	0.001	0.605
Day 5	6.00^a^	5.73^a^	5.64^b^	5.66^b^	0.25	0.033	0.154
Day 7	7.42^a^	7.52^a^	7.20^b^	7.18^b^	0.30	0.007	0.718

SEM, standard error of means; WHC, water holding capacity.

a,b Means in the same row with different superscripts differ (p<0.05).
